# Supplementary material for: In Silico Analysis of the Longevity and Timeline of Individual Germinal Center Reactions in a Primary Immune Response
Source: Cells. 2021 Jul 9;10(7):1736. doi: 10.3390/cells10071736 (PMC8306527; doi:10.3390/cells10071736)
Supplement: Supplementary file 1 [file cells-10-01736-s001.zip › cells-1234392-supplementary.pdf]

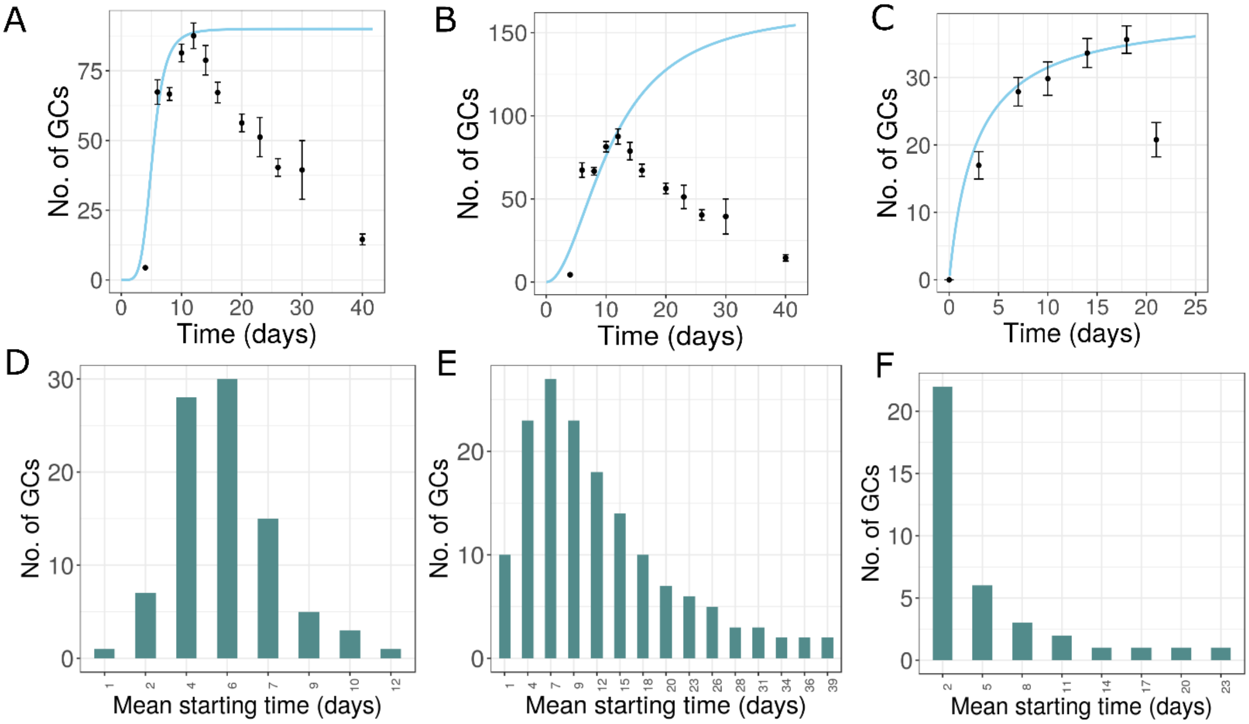

Supplementary Figure S1: Hill functions used to represent the appearance of new GCs (A-C) and the number of GCs represented by each simulated GC (D-F). The x axis in D-F shows the mean starting time of simulated GCs. Parameters of Hill function are (equation 1) $N_{\max} = 90, T = 125$ and n = 5 for A and D, $N_{\max} = 165, T = 260$ and n = 2 for B and E, $N_{\max} = 40, T = 65$ and n = 1 for C and F. A and D correspond to simulations in Figures 4-6, S2 and S3. B and E correspond to simulations with extended formation of new GCs in Figure 2. C and F correspond to Figures 7 and S4. GC: Germinal centre. Experimental data taken from [1] and [2].

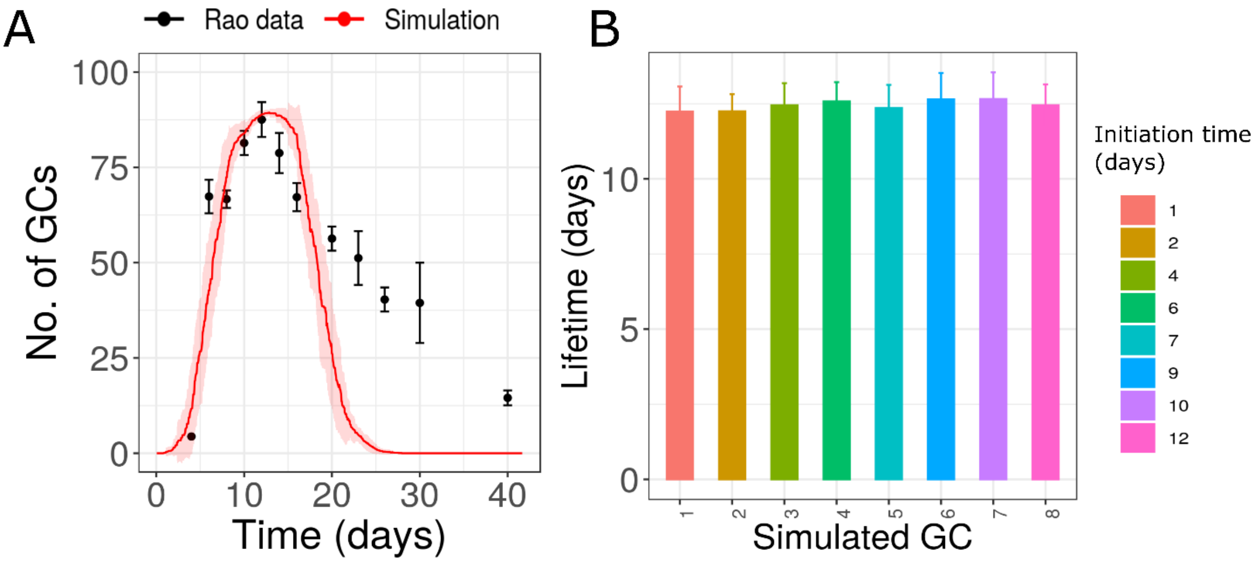

Supplementary Figure S2: Simulation of GCs with similar characteristics: Dynamics of number of GCs (A) and lifetime of simulated GCs (B). Color code in panel B represents initiation time of GCs. GC: Germinal centre. Experimental data taken from [1].

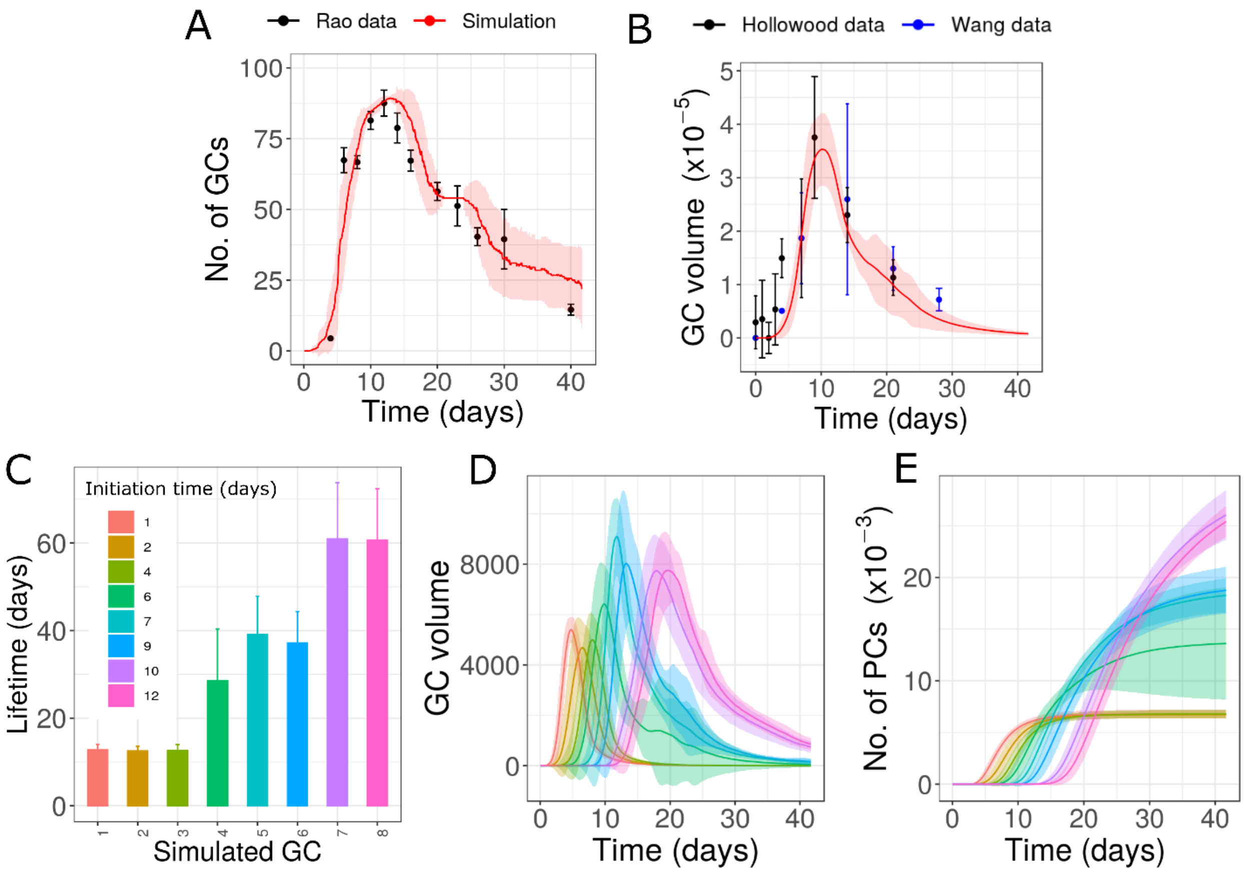

Supplementary Figure S3: Simulation of GCs with multiple epitopes and different founder cell specificities as in Figure 5 with a different set of founder cell specificities. A) Dynamics of number of GCs, B) Dynamics of total GC volume, C) Lifetime of simulated GCs D) GC volume of individual GCs simulated E) Plasma cells produced from simulated GCs. Color code in panels C, D and E represent initiation time of GCs. GC: Germinal centre; PC: Plasma cell. Experimental data taken from [1,3,4].

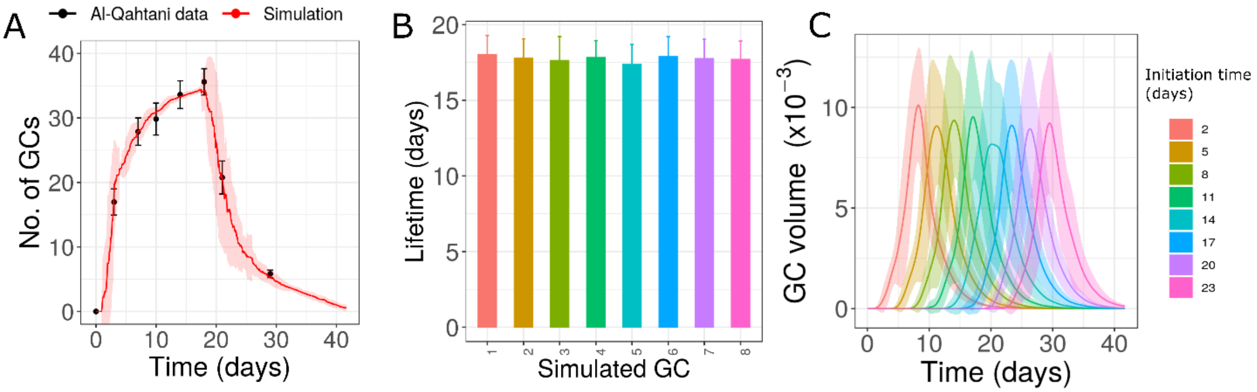

Supplementary Figure S4: Simulation of GCs with similar characteristics A) Dynamics of number of GCs in simulation compared to data, B) Lifetime of simulated GCs and C) Volume (measured as number of GC B cells) of simulated GCs. Color code in panels B and C represent initiation time of GCs. GC: Germinal centre. Experimental data taken from [2].

Supplementary Table S1. Parameter values used in the simulations of different hypothesis. GC: Germinal Centre; FDC: Follicular Dendritic Cell; Tfh: T follicular helper cell; Ag: Antigen.

Hypothesis	Reference parameter set	GCs with similar lifetime	Extended GC formation	Initiation time dependent Ag concentration	Founder specificity (Figure 5)	Founder specificity (Figure S3)	Initiation time independent variation in Ag conc.	GCs with similar lifetime (for NP-CGG data)	Initiation time dependent Ag concentration (for NP-CGG data)
Ag concentration (Portions per FDC)	3000	1500	800	20000 e -0.026t, where t is the GC initiation time	4650	5000	Gaussian distribution (mean 1300 and SD 1100)	3000	2000 e -0.01t, where t is the GC initiation time
Selection threshold by Tfh	0.5	0.1	0.1	0.5	0.5	0.5	0.5	0.1	0.5
Ag consumed during single interaction with FDC	1	2	2	1	1	1	1	2	1
No. of Tfh cells	250	250	300	250	250	250	250	250	250
No. of epitopes, position in shape space (proportion)	1,3333	1, 3333	1, 3333	1, 3333	3 epitopes<br>1111 (5 %),<br>5555 (25 %),<br>8888 (70 %)	3 epitopes<br>1111 (15 %),<br>5555 (30 %),<br>8888 (55 %)	1, 3333	1, 3333	1, 3333
Founder specificity (GC no. – distance from optimal position in shape space)	Random	Random	Random	Random	GC 1,2 – random;<br>GC 3 – dist. 1-3 (to 8888);<br>GC 4 – dist. 1-3 (to 5555);<br>GC 5 - 8 – dist. 1-3 (to 1111)	GC1 -3 – dist. 1-3 (to 1111);<br>GC4 – dist. 1-3 (to 5555);<br>GC5, 6 – dist. 1-3 (to 8888);<br>GC7, 8 – random	Random	Random	Random

References:

1. Rao, S.P.; Vora, K.A.; Manser, T. Differential Expression of the Inhibitory IgG Fc Receptor FcγRIIB on Germinal Center Cells: Implications for Selection of High-Affinity B Cells. *J. Immunol.* **2002**, *169*, 1859–1868, doi:10.4049/jimmunol.169.4.1859.

2. Al-Qahtani, A.; Xu, Z.; Zan, H.; Walsh, C.M.; Casali, P. A role for DRAK2 in the germinal center reaction and the antibody response. *Autoimmunity* **2008**, *41*, 341–352.

3. Wang, Y.; Carter, R.H. CD19 regulates B cell maturation, proliferation, and positive selection in the FDC zone of murine splenic germinal centers. *Immunity* **2005**, *22*, 749–761.

4. Hollowood, K.; Macartney, J. Cell kinetics of the germinal center reaction-a stathmokinetic study. *Eur. J. Immunol.* **1992**, *22*, 261–266.
